# SSR marker based analysis for identification and of genetic diversity of non-heading Chinese cabbage varieties

**DOI:** 10.3389/fpls.2023.1112748

**Published:** 2023-02-06

**Authors:** Jiwei Yin, Hong Zhao, Xingting Wu, Yingxue Ma, Jingli Zhang, Ying Li, Guirong Shao, Hairong Chen, Ruixi Han, Zhenjiang Xu

**Affiliations:** ^1^ College of Agriculture, South China Agricultural University, Guangzhou, China; ^2^ Institute for Agri-food Standards and Testing Technology, Shanghai Academy of Agricultural Sciences, Shanghai, China; ^3^ Development Center of Science and Technology, Ministry of Agriculture and Rural Affairs, Beijing, China; ^4^ State Key Laboratory of Crop Genetics and Germplasm Enhancement, College of Horticulture, Nanjing Agricultural University, Nanjing, China; ^5^ Research and development center, Fujian Jinpin Agricultural Technology Co., Ltd, Fuzhou, China

**Keywords:** non-heading Chinese cabbage, SSR, variety identification, DUS test, genetic diversity

## Abstract

As a widely cultivated vegetable in China and Southeast Asia, the breeding of non-heading Chinese cabbage (*Brassica campestris* ssp. *chinensis* Makino) is widespread; more than 400 varieties have been granted new plant variety rights (PVRs) in China. Distinctness is one of the key requirements for the granting of PVRs, and molecular markers are widely used as a robust supplementary method for similar variety selection in the distinctness test. Although many genome-wide molecular markers have been developed, they have not all been well used in variety identification and tests of distinctness of non-heading Chinese cabbage. In this study, by using 423 non-heading Chinese cabbage varieties collected from different regions of China, 287 simple sequence repeat (SSR) markers were screened for polymorphisms, and 23 core markers were finally selected. The polymorphic information content (PIC) values of the 23 SSR markers ranged from 0.555 to 0.911, with an average of 0.693, and the average number of alleles per marker was 13.65. Using these 23 SSR markers, 418 out of 423 varieties could be distinguished, with a discrimination rate of 99.994%. Field tests indicated that those undistinguished varieties were very similar and could be further distinguished by a few morphological characteristics. According to the clustering results, the 423 varieties could be divided into three groups: pak-choi, caitai, and tacai. The similarity coefficient between the SSR markers and morphological characteristics was moderate (0.53), and the efficiency of variety identification was significantly improved by using a combination of SSR markers and morphological characteristics.

## 1 Introduction

Non-heading Chinese cabbage (*Brassica campestris* ssp. *chinensis* Makino) is a subspecies of *Brassica* in the family Brassicaceae. It is usually diploid (2n = 20, AA) and comprises five types: pak-choi (var. *communis* Tesn et Lee), caitai (var. *tsai-tai* Hort), tacai (var. *rosularism* Tesn et Lee), taicai (var. *tai-tsai* Hort), and duotoucai (var. *multiceps* Hort) (Hou and Song, 2012). Originating in China, it has a long history of cultivation, with its leaves being the main product (Hou et al., 2020). Given its strong adaptability, short growth cycle, and rich nutritional value, the non-heading Chinese cabbage is widely planted not only in China, but also in Southeast Asia, Europe, and America, and is gradually becoming a global vegetable. Non-heading Chinese cabbage breeding is widespread in China. As of August 2022, the number of applications for plant variety rights (PVRs) of non-heading Chinese cabbage in China had reached 436. However, owing to the lack of outstanding inbred lines and germplasm resources, the genetic background of newly developed varieties of non-heading Chinese cabbage is becoming narrower, and variety identification is becoming more and more difficult.

A distinctness, uniformity, and stability (DUS) test is the key technical support for the granting of PVRs, in which the distinctness test is the key step. To assess distinctness, the candidate variety needs to be compared with any other commonly known varieties. To ensure the effectiveness and accuracy of the distinctness assessment, the construction of the database of commonly known varieties is very urgent and necessary. The effectiveness and accuracy of any distinctness assessment relies on the existence of a comprehensive and accurate database of commonly known varieties. The currently available database is based on the morphological characterization of commonly known varieties, which, although accurate and scientifically robust, also has several limitations, being slow, expensive, resource intensive, and time-consuming (Liu et al., 2012). In addition, as morphological characteristics are easily affected by environmental factors, such as temperature, light, and fertilizer application, and data collected in different ecological places or in different seasons may be quite different, which may cause errors when screening for similar varieties using the distinctness test. DNA molecular marker technology can directly detect differences on a DNA level among varieties; this technology is not easily affected by environmental conditions, does not require field planting, and is fast and efficient. It has been widely used in variety identification and is recommended as a supplementary method to construct a variety database for variety management, especially for screening for similar varieties using the distinctness test developed by the UPOV (International Union for the Protection of New Varieties of Plants). In contrast to other molecular markers, simple sequence repeat (SSR) markers have the advantages of clear loci, simple technology, and reliable detection results, and are recommended as one of the preferred markers for plant variety identification and database construction by UPOV (Zhou et al., 2020). SSR markers have been widely used in identifying Brassicaceae Burnett vegetables, such as Chinese cabbage, *Brassica* juncea, broccoli, and cauliflower (Zhan et al., 2014; Yan et al., 2021; Chu et al., 2020), and are also used in the identification and genetic diversity assessment of non-heading Chinese cabbage (Wang et al., 2008; Liu et al., 2014; Yu et al., 2014; Liu et al., 2021). However, in previous studies in non-heading Chinese cabbage, the SSR markers selected were comparatively low in polymorphism and could not meet the needs of large-scale variety identification (Li, 2010; Liu et al., 2014). The varieties that could be identified were usually limited to one or a few types, and did not cover all five types of non-heading Chinese cabbage; in addition, SSR data were mainly obtained by polyacrylamide gel electrophoresis, which was not conducive to genotyping and data-sharing. Therefore, it is necessary to establish a high-throughput SSR molecular identification system with a strong discriminatory ability that covers various types of non-heading Chinese cabbage, and which may provide a robust technical support for screening similar varieties using distinctness tests, identification of variety authenticity, and protection of PVRs.

In this study, by using non-heading Chinese cabbage varieties covering all the five types from all the main production areas in China, we tried to select a core set of SSR markers with high levels of polymorphism and strong discriminatory ability suitable for both polyacrylamide gel electrophoresis and capillary electrophoresis platforms. Based on the core SSR markers, an SSR fingerprint database could be constructed to provide a powerful support for similar variety screening of distinctness tests and variety identification of non-heading Chinese cabbage varieties.

## 2 Materials and methods

### 2.1 Plant materials and DNA extraction

**Figure 1:**
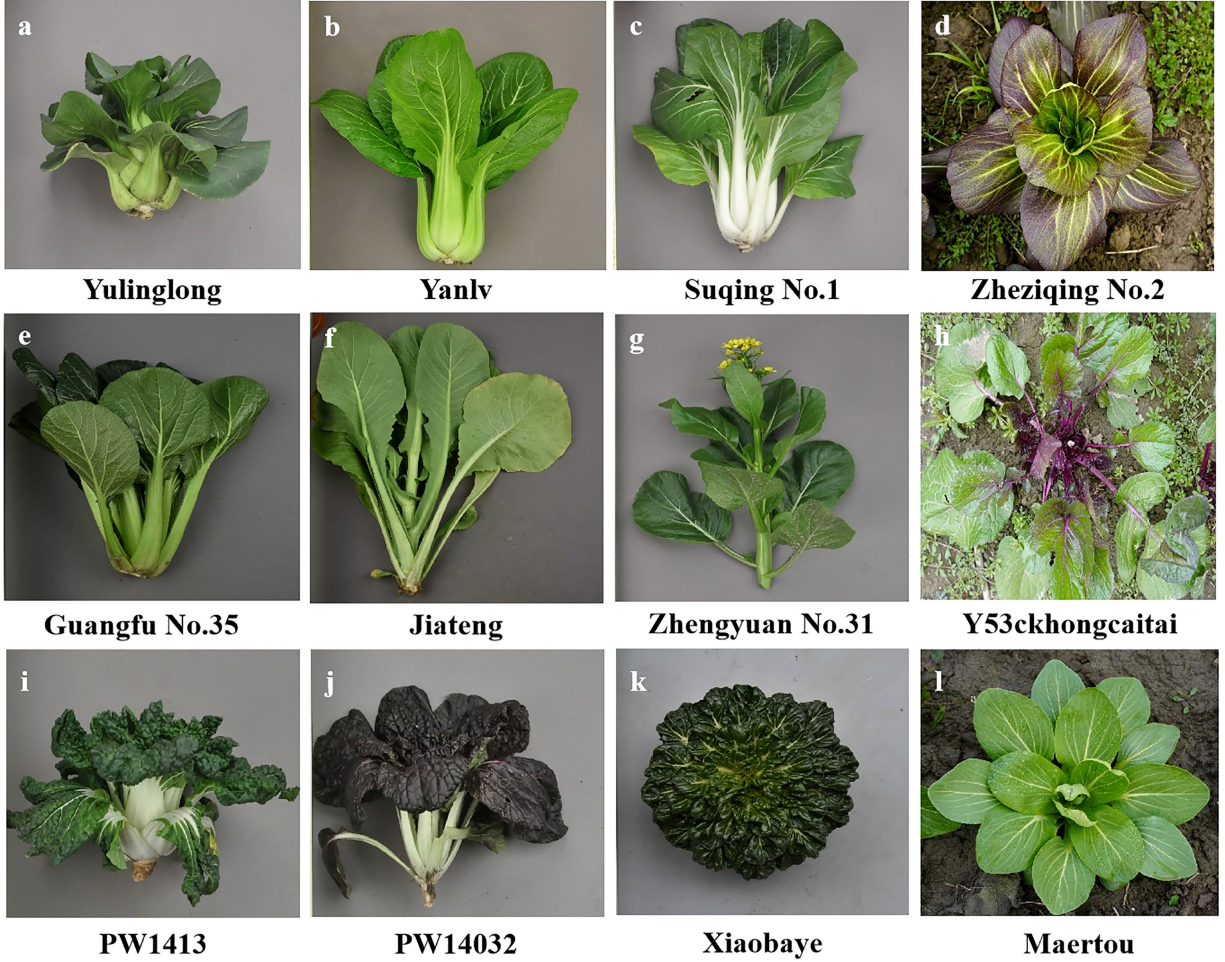
Different types of non-heading Chinese cabbage varieties: **(A–D)** pak-choi; **(E–H)** caitai; **(I–K)** tacai; and **(L)** duotoucai.

Non-heading Chinese cabbage has rich morphological diversity and exhibits significant differences among variety types (**Figure 1**). A total of 423 non-heading Chinese cabbage varieties covering five subspecies, pak-choi, caitai, tacai, taicai, and duotoucai (**Table S1**), were collected in this study, among which two varieties were from northeast China, 40 were from north and central China, 36 were from south China, 335 were from east China, and 10 from Japan. In addition, 21 varieties with diverse morphological characteristics were used for first-round SSR marker screening (**Table S2**). The young leaves from 30 individual plants were collected for total genomic DNA extraction, using the cetyltrimethylammonium bromide (CTAB) method, as previously described (Tang et al., 2007).

### 2.2 SSR-PCR reaction

A total of 287 markers were selected from previous studies (Lowe et al., 2002; Wang et al., 2008; Ban, 2009; Cheng et al., 2009; Li, 2010; Liu et al., 2014; Song et al., 2015; Chen et al., 2017; Liu, 2017; Li et al., 2018; He et al., 2021) (**Table S3**). The selected SSR markers were labelled with 6-FAM (6-carboxyfluorescein), HEX (hexachlorofluorescein), ROX (6-carboxyl-X-rhodamine; passive reference dye), and TAMRA (5-carboxytetramethylrhodamine) fluorescent dyes at the 5′ end of the forward primer. The total volume of the polymerase chain reaction (PCR) was 20 µL, with a dNTP concentration of 0.20 mmol/L, and concentrations of forward and reverse primers of 0.25 µmol/L, 0.05 U/µL of *Taq* total genomic DNA polymerase, 1 × PCR buffer (containing Mg^2+^, 2.5 mmol/l), and 50 ng/µL of DNA, and with the addition of double-distilled H_2_O up to a total of 20 µL. The PCR reaction conditions were as follows: pre-denaturation at 94°C for 5 minutes; denaturation at 94°C for 30 seconds, annealing at 55°C for 30 seconds, extension at 72°C for 45 seconds, for a total of 35 cycles; followed by extension at 72°C for 10 minutes; and then storage of the PCR reaction at 4°C.

### 2.3 Detection of PCR amplification products

During the first round of primer screening, primers were selected and detected by 6% polyacrylamide gel electrophoresis (PAGE) (Bao, 2015), with a constant power of 80 W; 2 µL of PCR product was added to each sample hole, and silver staining was performed after electrophoresis for 1–1.5 hours. Then the primers screened during the first round were labeled with different fluorescent dyes and were further screened and detected by a DNA analyzer (ABI3730).

### 2.4 Morphological evaluation

From December 2021 to March 2022, 423 non-heading Chinese cabbage varieties were planted at the Shanghai DUS testing base. In accordance with non-heading Chinese cabbage DUS test guidelines (http://www.nybkjfzzx.cn), 30 morphological characteristics were investigated (**Table 1**): four qualitative characteristics, 11 pseudo-qualitative characteristics, and 15 quantitative characteristics. The Shannon–Wiener diversity index of morphological characteristics was calculated as *H*′ = –∑(Pi) (lnPi), where Pi is the proportion of individuals to total individuals of this species. The 'Pi' is an explanation of the formula, and the specific number of individuals depends on the expression state of the characteristics. were assigned a code from 1 to 9. For each characteristic of a variety, the expressed state was coded as 1 and the non-expressed state was coded as 0. The programming language R was used for 0 or 1 data format conversion, to build a 0/1 data matrix.

**Table 1:** Details of morphological characteristics investigated in 423 non-heading Chinese cabbage varieties.

No	Characteristics	Type	Expression State (code)	H'	No	Characteristics	Type	Characteristic expression (code)	H'
1	Tiller	QL	absent(1); present(9)	0.04	16	Plant habit	QN	erect(1); erect to semi-erect(2);semi-erect(3); semi-erect to collapse(4); collapse(5)	0.98
2	Leaf hairiness	QL	absent(1)	0	17	Girdling	QN	absent(1); weak(2); medium(3); strong(4)	1.20
3	Leaf vein clarity	QL	weak(1); strong(2)	0.22	18	Plant height	QN	very low(1); very low to low(2); low(3); low to medium(4); medium(5); medium to high(6);high(7); high to very high(8); very high(9)	1.90
4	Inflorescence stem wax powders	QL	absent(1); present(9)	0.43	19	Plumpness of cabbage	QN	loose(1); medium(2); hard(3)	0.94
5	Seed coat color	PQ	yellow(1); brown(2); dark brown(3)	0.63	20	Leaf length	QN	very short(1); very short to short(2); short(3); short to medium(4); medium(5); medium to long(6); long(7); long to very long(8); very long(9)	1.90
6	Cotyledon color	PQ	light green(2); medium green(3); dark green(4); purple(5)	0.85	21	Leaf width	QN	very narrow(1); very narrow to narrow(2); narrow(3); narrow to medium(4); medium(5); medium to broad(6); broad(7); broad to very broad(8); very broad(9)	1.98
7	Leaf type	PQ	platy(1); divided leaf(2)	0.06	22	Leaf undulation of margin	QN	absent(1); 2(very weak)	1.39
8	Leaf shape	PQ	lanceolate (1); oval (2); elliptic(4); round oval(5); near round(6)	1.51	23	Leaf degree of blistering	QN	absent(1); very weak(2); weak(3); weak to medium(4); medium(5); medium to strong(6); strong(7); strong to very strong(8); very strong(9)	1.37
9	Leaf apex	PQ	blunt tip(1); circle(3); broad circle(4)	0.73	24	Leaf glossiness	QN	absent(1); weak(2); strong(3)	0.68
10	Leaf color	PQ	yellow green(1); light green(2); medium green(3); dark green(4); deep green(5); purple-red(6); purple(7)	1.34	25	Leaf number	QN	very less to less(2); less(3); less to medium(4); medium(5); medium to more(6); more(7); more to very more(8); very more(9)	1.77
11	Leaf margin features	PQ	inward(1); flat(2); outward(3)	0.42	26	Petiole thickness	QN	very thin(1); very thin to thin(2); thin(3); thin to medium(4); medium(5); medium to thick(6); thick(7); thick to very thick(8); very thick(9)	1.97
12	Petiole shape in horizontal section	PQ	subcircular(1); crescent(2); flat(3)	0.11	27	Petiole length	QN	very short to short(2); short(3); short to medium(4); medium(5); medium to long(6); long(7); long to very long(8); very long(9)	1.55
13	Petiole color	PQ	white(1); green white(2); light green(3); medium green(4); dark green(5); purple(6)	1.28	28	Petiole width	QN	very narrow(1); very narrow to narrow(2); narrow(3); narrow to medium(4); medium(5); medium to broad(6); broad(7); broad to very broad(8); very broad(9)	1.79
14	Inflorescence stem color	PQ	green(2); light green(3)	0.13	29	Bolting period	QN	very early to early(2); early(3); early to medium(4); medium(5); medium to late(6); late(7); late to very late(8); very late(9)	1.40
15	Flower color	PQ	white(1); light yellow(2); yellow(3); orange red(4)	0.38	30	Axillary bud generation ability	QN	absent or very weak(1); very weak to weak(2); weak(3); weak to medium(4); medium(5); medium to strong(6); strong(7); strong to very strong(8); very strong(9)	2.01

QL, qualitative characteristic; QN, quantitative characteristic; PQ, pseudo-qualitative characteristic; H′, Shannon–Wiener diversity index. Calculating by H′ = –∑(Pi) (lnPi), where Pi is the proportion of individuals to total individuals of this species.

### 2.5 Data analysis

Raw electrophoresis data were read by SSR Analyzer V1.2.6 software (Wang et al., 2018). The genetic distance between different varieties was calculated by PowerMarker V3.25 software (Liu and Muse, 2005), and the unweighted pair group method with arithmetic means (UPGMA) clustering map based on Nei’s genetic distance was constructed using MEGA5.0 software (Kumar et al., 2004). Genetic diversity parameters, including minor allele frequency (MAF), observed number of alleles (Na), observed heterozygosity (Ho), expected heterozygosity (He), polymorphic information content (PIC), and fixation index (Fst), were calculated using GenAlEx 6.51 software (Peakall and Smouse, 2012), which was also used for principal component analysis (PCA) and analysis of molecular variance (AMOVA). Combining morphological and molecular data, NTSYS2.11 software was used for genetic similarity analysis (James, 1987). Using qualitative data in the similarity module, the original 0/1 matrix generated by the morphological characteristic code and genotype data was adopted to calculate the genetic similarity (GS). The Mantel test was used to confirm the correlation between the similarity coefficient matrix generated from the morphological data and the SSR genotype data. Structure 2.34 software was used to analyze the population genetic structure from different regions of China (Falush et al., 2007). Assuming that the population number *K* was 1–10 and was tested one by one, each *K*-value was estimated to be repeated 20 times: 5,000 iterations were not counted and the MCMC (Markov chain Monte Carlo) value was 50,000. The average value of ln*P* (*D*) was used for population estimation, the optimal population number was determined by the maximum likelihood method, and the corresponding *K*-value was calculated. Finally, we used NTSYS2.11 software to test the similarity of the phenotypic data of 14 varieties (five candidate varieties, with their corresponding similar varieties provided by applicants, and those screened by the SSR fingerprint database in this study).

## 3 Results

### 3.1 Establishment of variety identification system for non-heading Chinese cabbage

#### 3.1.1 Core primer screening and polymorphism analysis

**Figure 2:**
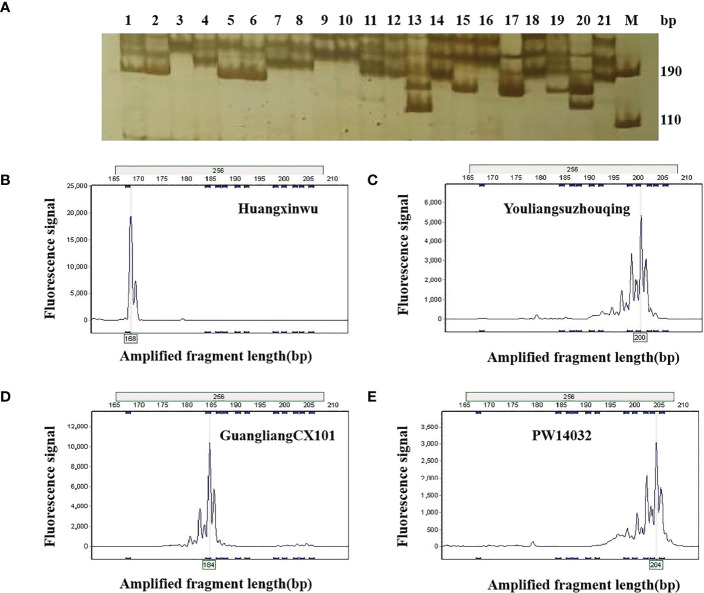
Allelic variation in 21 non-heading Chinese cabbage varieties using by primer SSR256. **(A)** Allelic variation in 21 varieties using PAGE. **M**, DNA marker; the number 1–21 in Figure 1a corresponds to the variety number in **Table S2**. **(B–E)** show the allelic variations in varieties 17, 9, 14, and 16 by fluorescence capillary electrophoresis. PAGE, polyacrylamide gel electrophoresis.

During the first round of primer screening, 21 representative varieties were used for PCR amplification, and 6% PAGE was used for electrophoresis (**Figure 2A**). As a result, 57 pairs of primers with high levels of polymorphism were screened. During the second round of primer screening, fluorescent dyes were labelled at the 5′ end of each of the 57 pairs of primers, and the fluorescent markers were used to amplify 96 varieties by capillary electrophoresis (**Figures 2B–E**). Based on the criteria of stable and simple fluorescence peak, low missing rate, high levels of polymorphism, and even distribution on chromosomes, 23 pairs of primers were finally selected as core primers, with the size of alleles ranging from 99 bp (SSR221) to 355 bp (SSR227). Detailed information on those primers is provided in **Table 2**.

**Table 2:** Chromosome distribution and allelic variation range of the 23 primer sequences studied.

No.	Primer number	Sequence (5′→3′)	Chromosomal position	Allelic variation range (bp)
1	SSR101	F: TGGAGTGTTTGTTGTAAGCTCAA R: TTCGGGATGAGAGTTCCAAG	5	188–227
2	SSR125	F: TGCTCTTTGACACGTGCTATC R: AGAGGAGAGAAGGGGAGAGG	1	110–139
3	SSR136	F: TGATCACTGGGGTCCATTTA R: CTGCGTCGAAGTTAGAGACG	2	153–203
4	SSR138	F: TGCGTGCGGATTATCATCTA R: GGACGTAAACTTAGCACGATTC	2	160–174
5	SSR192	F: TAATCGCGATCTGGATTCAC R: ATCAGAACAGCGACGAGGTC	5	114–162
6	SSR198	F: GGTCAGGTGCTACTCAGACTCC R: TTGAAGAGGATCCACCAAAAG	3	276–314
7	SSR206	F: TGTCAGTGTGTCCACTTCGC R: AAGAGAAACCCAATAAAGTAGAACC	8	124–207
8	SSR207	F: TCAGCCTACCAACGAGTCATAA R: AAGGTCTCATACGATGGGAGTG	6	144–213
9	SSR22	F: ATGCACAGAGGAAGAAACCG R: GGGGATGAAGAAGAAGCAGA	1	155–191
10	SSR221	F: GTTCTCAAAGGGAAACCGAAAAACA R: GAGTTGGCCAGAGATTTACATGCGT	4	99–178
11	SSR222	F: CAAGAGCAAGTTTGAAACAAACGAT R: CATCAGTTCTTGATATGCTAGGTGA	6	175–280
12	SSR227	F: TTCCACCTCTCTGCTCCAAC R: ATGCGTGAGCGAGGATAACT	2	271–355
13	SSR228	F: GGAGTCCACTTCATGGAGGA R: CTCTTGCTCGTAGGTTTCCG	8	233–274
14	SSR229	F: TCAGTCACAAAAAGTCAACTCAAA R: ACGGAGTAGGAGTTGGGAGG	9	114–148
15	SSR238	F: TTTGACATCGTGCAATGCTA R: TTGGGCTGGTCCTGAAGATA	3	278–325
16	SSR247	F: GGTCCATTCCTTTTTGCATCTG R: CATGGCAAGGGGTAACAAACAT	7	128–154
17	SSR256	F: GGAGCCAGGAGAGAAGAAGG R: CCCAAAACTTCCAAGAAAAGC	3	168–206
18	SSR266	F: TCGGATTTGCATGTTCCTGA R: CCGATACACAACCAGCCAACT	7	187–305
19	SSR283	F: CCAACACCAAATCGCATAATC R: GGAGCTCCCACCTACAGTTTC	10	163–182
20	SSR45	F: GATTTGGGCCATTTGGATTA R: TTGAGCATTGTTCCCAGACA	4	206–230
21	SSR56	F: GTTAAGTTCGAACGCGAAGG R: GATCGGGGAAAATTAGGGAA	9	241–272
22	SSR66	F: ATTCAAAGACAAAGGAATGCCTGAG R: GTTTCTTTGATCCTGTCGAATGGCATTAATAAA	6	123–144
23	SSR90	F: TGCCTTTGTGTTCAGCTCAC R: CCCAAACGCTTTTGACACAT	10	202–211

bp, base pair; F, forward; R, reverse.

Using 23 pairs of primers, 423 non-heading Chinese cabbage varieties were detected and a total of 314 alleles were obtained, with an average of 13.65 alleles per marker (**Table 3**). The variation range of MAF was 0.209 (SSR222) to 0.611 (SSR101), with an average of 0.419; the Ho amplitude ranged from 0.322 (SSR101) to 0.732 (SSR256), with an average of 0.530; the He amplitude was between 0.590 (SSR125) and 0.916 (SSR222); the PIC value ranged from 0.555 (SSR56) to 0.911 (SSR222), greater than 0.5, indicating high levels of polymorphism of all 23 markers; and the Fst of each molecular marker ranged from 0.045 (SSR221) to 0.547 (SSR266), with an average of 0.270. The above parameters showed that the 23 markers selected were high in polymorphism and could be used for genetic diversity detection among non-heading Chinese cabbage varieties, variety identification, and similar variety screening for the distinctness test.

**Table 3:** Genetic parameters of the 23 SSR markers.

Marker	MAF	Na	Ho	He	PIC	Fst
SSR101	0.611	10	0.322	0.596	0.572	0.460
SSR125	0.610	14	0.386	0.590	0.561	0.346
SSR136	0.380	7	0.531	0.750	0.712	0.292
SSR138	0.335	8	0.723	0.794	0.767	0.089
SSR192	0.454	20	0.617	0.753	0.735	0.181
SSR198	0.599	12	0.490	0.616	0.598	0.204
SSR206	0.365	26	0.677	0.826	0.814	0.181
SSR207	0.527	6	0.336	0.618	0.557	0.455
SSR22	0.429	10	0.560	0.732	0.696	0.235
SSR221	0.490	16	0.693	0.725	0.708	0.045
SSR222	0.209	40	0.601	0.916	0.911	0.344
SSR227	0.233	23	0.461	0.850	0.834	0.458
SSR228	0.498	13	0.501	0.686	0.651	0.269
SSR229	0.474	9	0.546	0.654	0.596	0.164
SSR238	0.413	12	0.467	0.760	0.733	0.386
SSR247	0.346	7	0.579	0.701	0.644	0.174
SSR256	0.177	16	0.732	0.864	0.849	0.153
SSR266	0.415	30	0.356	0.787	0.771	0.547
SSR283	0.388	6	0.556	0.725	0.678	0.234
SSR45	0.456	7	0.414	0.671	0.614	0.383
SSR56	0.495	7	0.513	0.624	0.555	0.177
SSR66	0.283	10	0.551	0.780	0.744	0.293
SSR90	0.452	5	0.586	0.690	0.640	0.151
Mean	0.419	13.6	0.530	0.726	0.693	0.270

MAF, minor allele frequency; Na, observed number of alleles; Ho, observed heterozygosity; He, expected heterozygosity; PIC, polymorphic information content; Fst, fixation index.

#### 3.1.2 Allelic sites calibration and database construction

According to the original capillary electrophoresis data, different allelic sites were named, and each allele’s corresponding reference varieties were selected to calibrate systematic errors among different experimental batches or detection platforms. The size of allelic sites corresponding to each primer and the corresponding reference varieties are listed in **Table S4**.

Based on the allelic sites data detected on the 423 non-heading Chinese cabbage varieties, the DNA molecular database was successfully constructed using SSR Analyzer V1.2.6 software. To improve the efficiency of database construction, 23 pairs of fluorescent primers were further divided into five groups (**Table 4**) according to the fluorescent color and amplified fragment size. Primers in each group could be mixed for multiple fluorescent capillary electrophoresis.

**Table 4:** Grouping of 23 core primers according to different fluorescent-labelled colors.

Group Label	1	2	3	4	5
6-FAM	SSR125(110–139)	SSR138(150–174)	SSR256(164–206)	SSR221(99–178)	SSR136(153–203)
	SSR283(163–182)	SSR45(206–228)	SSR56(241–270)		SSR198(278–314)	
	SSR228(233–274)	SSR227(271–355)			
ROX	SSR66(123–148)	SSR101(197–225)	SSR247(128–149)	SSR90(174–211)	SSR192(116–158)
	SSR266(188–305)	SSR238(278–325)			SSR222(175–227)	
TAMRA		SSR229(114–148)	SSR22(155–194)	SSR206(124–207)	
HEX	SSR207(144–214)				

The selected SSR markers were labelled with 6-FAM, (6-carboxyfluorescein); HEX, (hexachlorofluorescein); ROX, (6-carboxyl-X-rhodamine; passive reference dye); or TAMRA, (5-carboxytetramethylrhodamine) fluorescent dyes at the 5′ end of the forward primer.

#### 3.1.3 Discrimination power of core primers

**Figure 3:**
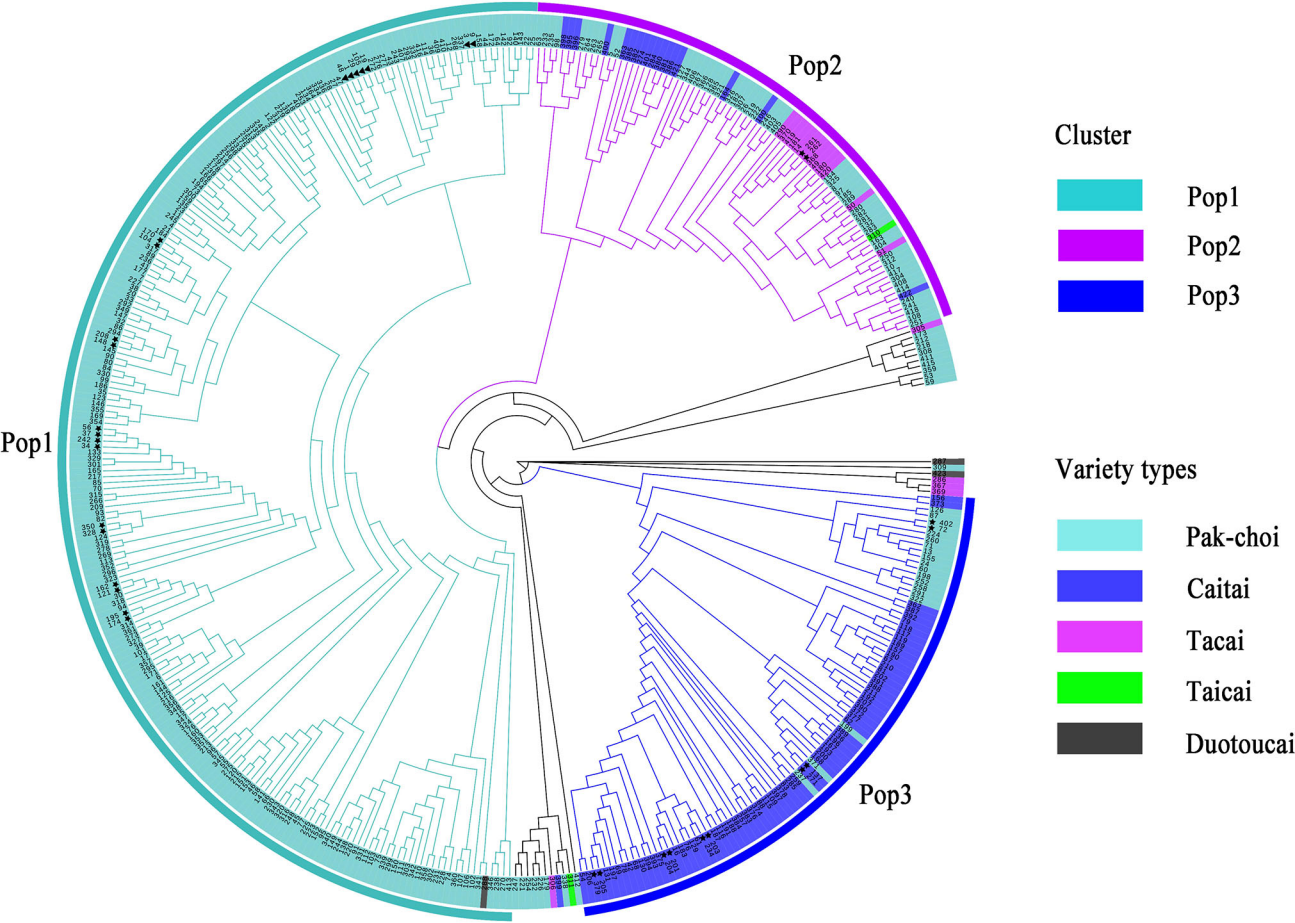
Cluster of 423 non-heading Chinese cabbage varieties based on Nei’s distance of 23 simple sequence repeat (SSR) markers. ▲ represents two varieties with zero difference in the number of alleles; ★ represents two varieties with one difference in the number of alleles.

**Figure 4:**
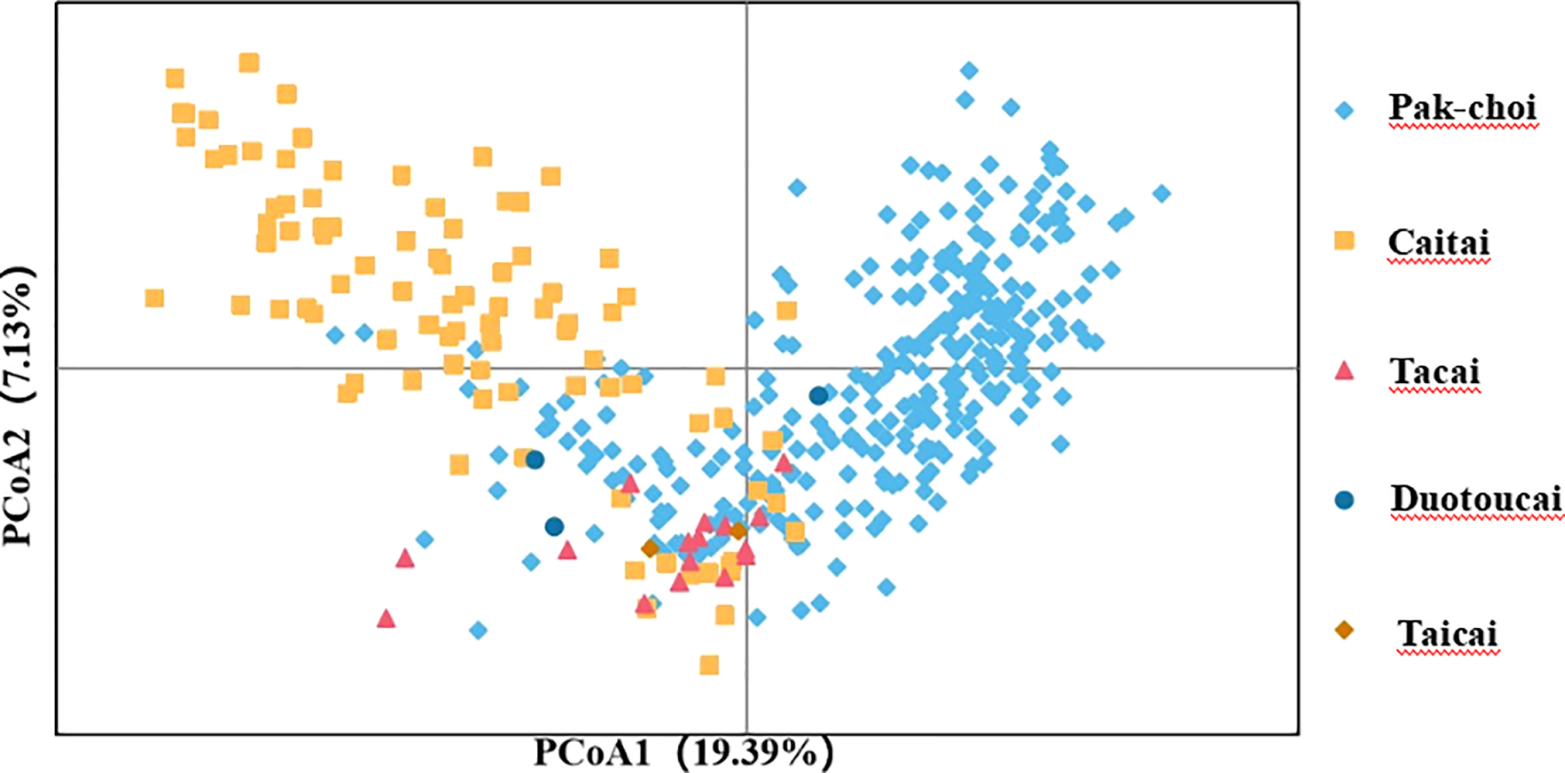
Principal component analysis (PCA) of 423 non-heading Chinese cabbage varieties based on 23 simple sequence repeat (SSR) markers. PCA results explained 19.39% and 7.13%, respectively, of the total variance by calculating the first two principal components.

To assess the accuracy and efficiency of core primers in distinguishing varieties, the 423 non-heading Chinese cabbage varieties were clustered based on the Nei’s distance of 23 SSR markers (**Figure 3**). The clustering results showed that 418 out of the 423 varieties combinations could be distinguished by the 23 core primers, and that five groups could not be distinguished,because the genetic distance between varieties in each group was close to zero. By using the formula [(423 * 422)/2 – 5]/(423 * 422)/2, the distinguishing rate for the 23 core SSR markers in 423 varieties was calculated to be 99.994%. By clustering, the 423 varieties could be divided into three main groups. In Pop1(n=226), besides duotoucais, the other varieties were pak-chois; Pop2(n=83) contained 52 pak-chois, 13 tacais, 17 caitais, and one taicai; and Pop3(n=93) comprised 19 pak-chois and 74 caitais. In addition, some local varieties were clustered separately, such as Shangwudong (412), Paopaoqing (286), and Xiangqingcai (308). Similarly, we conducted PCA to verify the clustering results, and principal coordinates 1 and 2 accounted for 19.39% and 7.13%, respectively, of the variation in the site information data (**Figure 4**). The AMOVA results showed that 66% of the variation came from within individuals. The genetic variation among individuals was greater than that among populations (**Table S5**). The fixation index (Fst) value was 0.134 (*p* < 0.001), indicating a high level of genetic differentiation among populations.

### 3.2 Population structure analysis of the tested varieties

**Figure 5:**
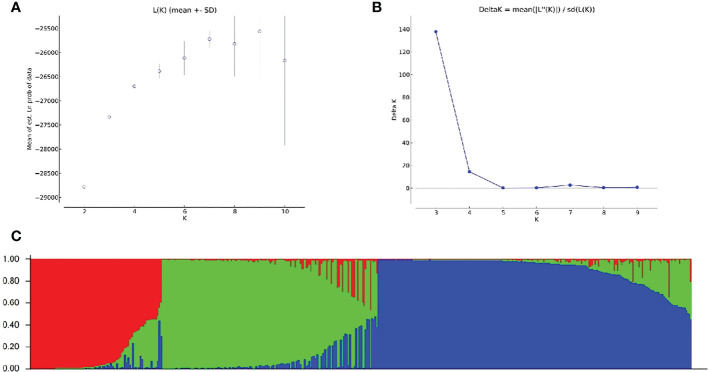
Population structure analysis of 423 non-heading Chinese cabbage varieties based on 23 simple sequence repeat (SSR) markers. **(A)** The mean value of ln*P* (D) was used to estimate the population structure, and the range of *K*-values was 1–10. **(B)** Using the curve of Δ*K* obtained by ln*P* (D), the optimal *K*-value was determined to be 3. **(C)** The 423 non-heading Chinese cabbage varieties studied clustered in three subgroups (subgroup I, red; subgroup II, green; and subgroup III, blue). Each histogram represents a variety in which different colors represent the estimated component coefficients using *Q*-values.

To explore the population distribution characteristics of non-heading Chinese cabbage, the population structure of 423 varieties was analyzed using the genotype data. The results showed that, for *K* = 1–10, the value of ln*P* (*D*) increased with the increase in *K*-value (**Figure 5A**). A population structure distribution map based on Δ*k* was constructed (**Figure 5B**), and the 423 varieties could be divided into three subgroups (**Figure 5C**). There were 84 varieties in subgroup I, from east China (*n* = 70), south China (*n* = 6), north China (*n* = 1), central China (*n* = 4), and Japan (*n* = 3); 138 varieties in subgroup II, from east China (*n* = 113), south China (n = 10), north China (*n* = 7), central China (*n* = 5), and Japan (*n* = 3); and 201 varieties in subgroup III, from east China (*n* = 152), south China (*n* = 20), north China (*n* = 14), central China *(n* = 9), northeast China (*n* = 2), and Japan (*n* = 4) (**Figure 5C**).

The population structure analysis showed that most genetic differences among non-heading Chinese cabbage varieties could be attributed to the geographical origins of the varieties. Varieties in subgroup I were mainly from east China, and varieties from east China also accounted for a large proportion of the other two subgroups; the caitai varieties were mainly from south China and clustered in subgroup II; and varieties from north China and northeast China were mainly clustered in subgroup III.

### 3.3 Correlation analysis between SSR markers and morphological characteristics

**Figure 6:**
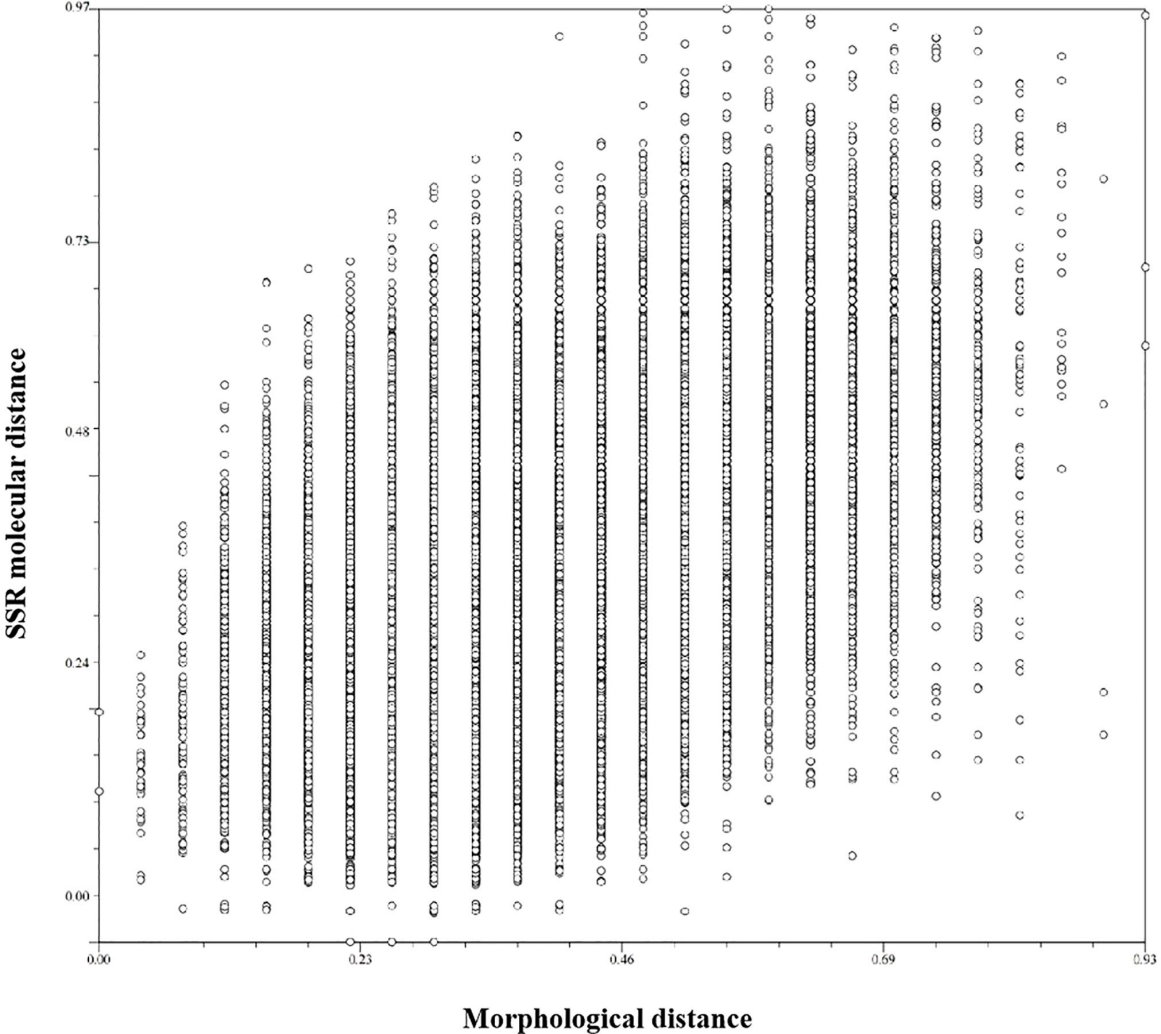
Comparison of morphological distance and molecular distance of 423 non-heading Chinese cabbage varieties. The abscissa is morphological distance, and the ordinate is molecular distance. The similarity coefficient is 0.53.

Descriptive statistics were based on 30 phenotypic characteristics of 423 non-heading Chinese cabbage varieties. The Shannon–Wiener diversity index of 30 characteristics ranged from 0 to 2.01, with an average of 1.03. In order to understand the relationship between SSR markers and morphological characteristics, the data from 30 morphological characteristics (markers) and those from the 23 core primers were converted into the 0/1 format, and the similarity coefficient of the two markers was calculated. The results showed that the similarity coefficient of the morphological markers and the SSR markers was moderate (*r* = 0.53) (**Figure 6**). Therefore, combining morphological and SSR markers would be more helpful for identifying non-heading Chinese cabbage.

**Figure 7:**
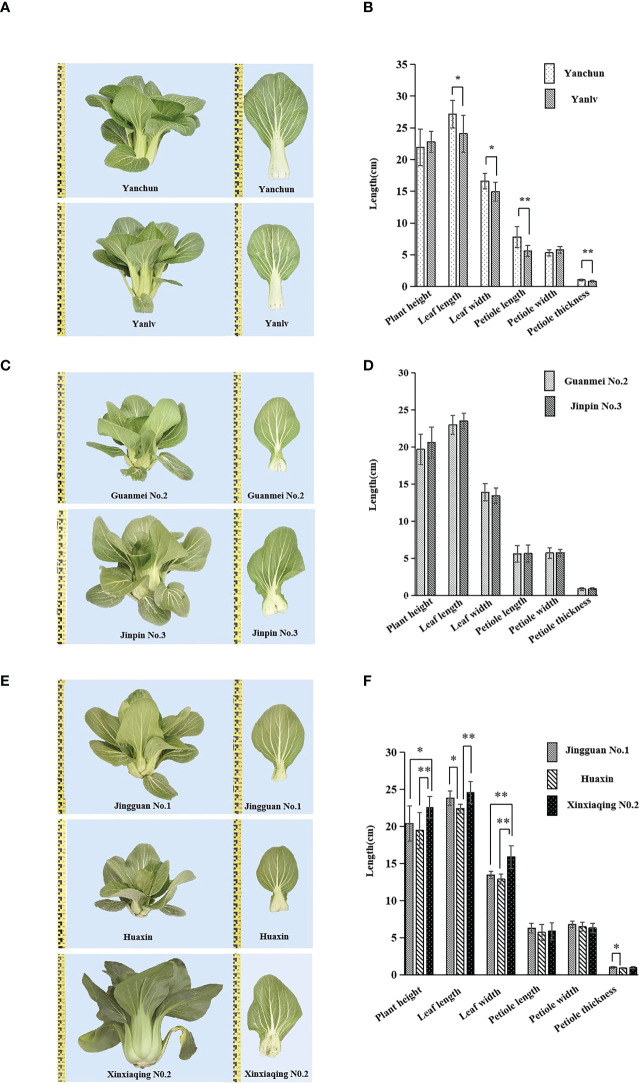
Phenotype comparison and quantitative characteristics ANOVA of five groups of varieties with no differences in simple sequence repeat (SSR) markers: ‘Yanchun’ and ‘Yanlv’ **(A, B)**; ‘Guanmei No. 2’ and ‘Jinpin No. 3’ **(C, D)**; ‘Jingguan No. 1’ and ‘Huaxin’, ‘Jingguan No. 1’ and ‘Xinxiaqing No. 2’, and ‘Huaxin’ and ‘Xinxiaqing No. 2’ **(E, F)**. Ten plants were used for the analysis (*significant at *p *< 0.05; **highly significant at *p *< 0.01).

Five groups not distinguished by the 23 core markers, ‘Yanchun’ and ‘Yanlv’, ‘Guanmei No. 2’ and ‘Jinpin No. 3’, ‘Jingguan No. 1’ and ‘Huaxin’, ‘Jingguan No. 1’ and ‘Xinxiaqing No. 2’, and ‘Huaxin’ and ‘Xinxiaqing No. 2’, were further compared through a field growing test. The plants in each group were very similar (**Figure 7**), although in each group slight differences were found in some visually observed characteristics such as seed coat color, plumpness of cabbage, leaf margin undulation, or bubble degree (**Table S6**). The variance analysis of six quantitative characteristics also revealed the existence of some differences in leaf length, leaf width, petiole length, and petiole thickness in four group varieties, but not in the group comprising ‘Guanmei No. 2’ and ‘Jinpin No. 3’ (**Figure 7**). These results indicated a certain degree of consistency in the identification of varieties between SSR markers and morphological characteristics. The identification results based on morphological characteristics were more accurate and reliable than those based on SSR markers and, when used together with the molecular markers, could obviously improve identification efficiency.

### 3.4 Application of the SSR fingerprint database

**Figure 8:**
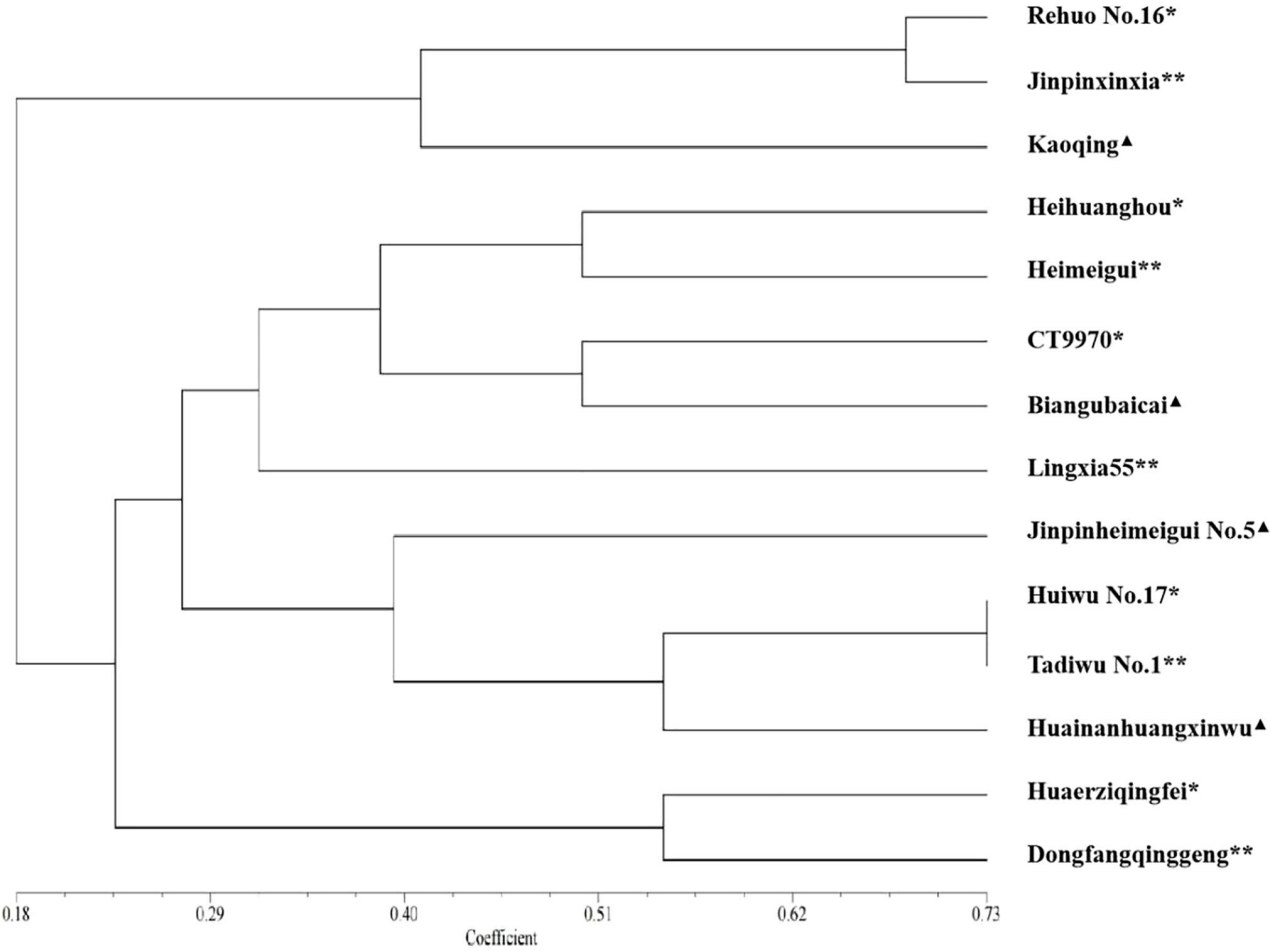
Similarity analysis of candidate varieties and their corresponding similar varieties based on 30 morphological characteristics. The scale at the bottom is the similarity coefficient. *: candidate varieties; **: varieties screened by SSR fingerprint database; ▲: the applicant submitted similar varieties.

In order to evaluate the application of the SSR fingerprint database in screening for similar varieties using the distinctness test, we selected five candidate varieties for which PVRs had been applied, and compared the similar varieties provided by the applicants (five varieties) with those screened by the SSR fingerprint database (five varieties) through the field planting test. (One of the varieties screened by the molecular fingerprint database was the same as the breeder provided, so there were 14 varieties.) The morphological characteristics comparison showed that four candidate varieties were similar to varieties screened by the SSR fingerprint database in this study (**Figure 8**). The morphological similarity between ‘Huiwu No. 17’ and ‘Tadiwu No. 1’ was 0.73, that between ‘Huaerziqingfei’ and ‘Dongfangqinggeng’ was 0.53, that between ‘Heihuanghou’ and ‘Heimeigui’ was 0.48, and that between ‘Rehuo No. 16’ and ‘Jinpinxinxia’ was 0.67.

As for candidate variety ‘CT9970’, it was more similar to the similar variety ‘Biangubaicai’ selected by the SSR fingerprint database than to the variety ‘Lingxia 55’ provided by its applicant. Breeding process analysis showed that ‘CT9970’ originated from ‘Biangubaicai’ and retained most of its morphological characteristics, whereas ‘Lingxia 55’ was the F_1_ generation of ‘CT9970’ and ‘CL45’ (caitai variety), and resulted in low levels of similarity with ‘CT9970’.

Thus, through morphological verification, the SSR fingerprint database can be used not only to screen similar varieties in the distinctness test, but also to preliminarily assess their genetic relationship.

## 4 Discussion

With the completion of whole-genome sequencing of non-heading Chinese cabbage, more SSR markers have been developed and utilized (Li et al., 2020). Because of the advantages of codominance and high levels of polymorphism, SSR markers provide an effective tool for studying the genetic diversity of non-heading Chinese cabbage (Li et al., 2021). In recent years, SSR markers have been widely used in predicting the genetic diversity and germplasm identification of non-heading Chinese cabbage germplasm resources (Han et al., 2008; Ma, 2015; Li et al., 2017; Li et al., 2018). In this study, by using 423 non-heading Chinese cabbage varieties with rich and diverse phenotypes, 23 pairs of SSR primers (out of 287 analyzed) with better performance than those used in previous studies (Xue et al., 2014; Yang et al., 2020) were identified (average PIC value of 0.693). This may be attributed to the large number of varieties collected, their rich genetic diversity, and the highly accurate capillary electrophoresis detection method used in this study.

Clustering results in this study showed that most of the 423 non-heading Chinese cabbage varieties fell into one of three main groups, pak-choi, caitai, and tacai, which was in line with the actual status of breeding and production. In the study of Ma et al. (2015), Pak-choi varieties are clustered with Caitai, and Tacai in different degrees, which was similar to the research results of this study. In addition, PCA in this study also showed that there was obvious interspecific crossing and extensive gene exchange between the pak-choi and caitai genetic backgrounds (**Figure 4**), but this phenomenon has been seldom mentioned in previous studies.

According to a previous study, purple is not completely dominant over green in the inheritance of non-heading Chinese cabbage, and the purple color largely depends on anthocyanin content (Zhu, 2017). However, we observed that the hybrid progeny of crosses between a green and a purple non-heading Chinese cabbage variety showed a distribution that was largely skewed towards the phenotype of purple parent, suggesting that all varieties of purple non-heading Chinese cabbage are likely to have the same genetic background.

The genetic diversity of non-heading Chinese cabbage was related to geographical origin (Wang et al., 2008; Liu et al., 2014). Population structure analysis in this study showed that, in east China, germplasm resources were more abundant and genetic diversity was greater, and the three provinces of Jiangsu, Zhejiang, and Fujian in east China had relatively independent genetic structures, which confirmed that non-heading Chinese cabbage in China might originate from the Jianghuai area (Cao et al., 1997).

Recent studies have shown that different varieties can be effectively distinguished and analyzed through complementary differences in morphological markers and molecular markers (Lee and Park, 2017). This complementary method is usually used in germplasm identification (Delfini et al., 2007; Haliloglu et al., 2022) and genetic diversity analysis (Guo et al., 2020; Chikh-Rouhou et al., 2021). Theoretically, one morphological characteristic would be usually regulated by multiple genes. In this study, only five groups could not be distinguished by the 23 core markers, and the field growing comparison test showed that varieties in each of the five groups were very similar but were still distinguishable by some visually observed or measured characteristic. Molecular markers correlated to a medium extent (*r* = 0.53) with morphological characteristics, which was higher than that in a previous study on peanuts (0.347) (Hong et al., 2021), but no functional molecular markers associated with morphological characteristics in non-heading Chinese cabbage were found in this study. Therefore, without enough functional markers, molecular markers cannot completely replace morphological markers, but a combination of both types of markers would be more accurate and efficient in variety identification and in similar variety screening for the distinctness test.

## 5 Conclusion

In this study, 23 out of 287 SSR markers were selected as the core markers, with an average PIC value of 0.693 and an average number of alleles of 13.65. Based on the 23 core markers, the SSR fingerprint database comprising 423 non-heading Chinese cabbage varieties was constructed, in which 418 out of the 423 varieties could be distinguished with a discrimination rate of 99.994%. The SSR fingerprint database constructed in this study could be used not only in the identification of varieties but also for similar varieties screening of distinctness test.

## Data availability statement

The original contributions presented in the study are included in the article/**Supplementary Material**. Further inquiries can be directed to the corresponding authors.

## Author contributions

ZX and RH designed and supervised this project. JY and HZ performed most of the experiments. XW, YM, and JZ carried out part of the experiments. JY, HZ, and XW participated in data analysis. JY, HZ, XW, RH, and ZX wrote the manuscript. YL, GS, and HC revised the manuscript. RH and ZX supervised the study and revised the manuscript. All authors contributed to the article and approved the submitted version.
